# Speech Perception and Preparation Are Supported by Distinct Neural Dynamics Across Development

**DOI:** 10.1162/NOL.a.219

**Published:** 2026-02-20

**Authors:** Yanni Liu, Amanda Hampton Wray, Soo-Eun Chang

**Affiliations:** Department of Psychiatry, University of Michigan, Ann Arbor, MI, USA; Department of Communication Science and Disorders, University of Pittsburgh, Pittsburgh, PA, USA; Department of Communication Disorders, Ewha Womans University, Seoul, Korea

**Keywords:** beta oscillations, developmental trajectories, EEG, speech processing

## Abstract

Speech development requires precise timing and sensorimotor integration, supported by neural oscillations that synchronize activity across auditory, motor, and cognitive circuits. Among speech-relevant frequency bands, beta oscillations (13–30 Hz) are critical for timing and coordination, supporting sensorimotor processing and speech preparation. Beta desynchronization (power decreases) is typically observed prior to movement, reflecting motor planning, and beta activity also supports cognitive functions such as attention and anticipation. Although age-related changes in beta power have been documented, its developmental trajectory during speech processing remains underexplored. Here, we compared beta power dynamics in 28 adults (mean age = 27.8 yr) and 50 children (mean age = 10.3 yr) during speech perception and production tasks using EEG. On each trial, participants received a visual cue indicating the condition (“Say,” “Hear,” or “See”), followed by rhythmic tones and a warning cue presented as a picture with its name (e.g., cat) in Say and Hear, but only as a picture in See. A Go cue then prompted participants to speak, listen, or maintain fixation. Beta power was analyzed in three time windows: postwarning cue (P1), pre-Go cue (P2), and post-Go cue (P3). Adults exhibited significant beta power decreases across all time windows, particularly in Say, indicating mature sensorimotor and cognitive integration. In contrast, children showed no significant condition effects and minimal beta reduction in P3. Beta modulation was negatively correlated with age in children, suggesting ongoing maturation of beta oscillations. These findings highlight key developmental differences in beta oscillations relevant to speech processing.

## INTRODUCTION

### Oscillatory Mechanisms and Motor Integration in Speech

Understanding the neural dynamics involved in speech processing is essential for uncovering the mechanisms that support fluent language development. One way to examine neural dynamics is through measuring neural oscillations in relevant frequency bands that provide scaffolding for timing and coordination of neural activity. Among the speech-relevant frequency band activities studied in previous research, oscillatory neural activities in the beta (13–30 Hz) and the alpha/mu (8–12 Hz) frequency bands have been reported as important for supporting interactions between cognitive, sensory, and motor systems (Cuevas & Bell, 2022; Jenson et al., 2018; Kilavik et al., 2013; Saltuklaroglu et al., 2018).

In the speech domain, alpha/mu oscillations have been implicated in attentional modulation and sensory gating, supporting selective attention to relevant auditory speech cues under adverse listening conditions (Obleser & Weisz, 2012; Saltuklaroglu et al., 2018). Mu rhythm suppression over sensorimotor cortices has also been linked to articulatory simulation and auditory–motor mapping, suggesting that alpha/mu oscillations contribute not only to sensory gating but also to the integration of perceptual and motor processes during speech (Bowers et al., 2013; Jenson et al., 2014). While alpha/mu oscillations are generally associated with attentional modulation and sensory gating, beta oscillations are more strongly linked to motor preparation, top-down control, and predictive timing—key components of speech production and fluent communication (Engel & Fries, 2010; Hickok, 2012; Kilavik et al., 2013; Saltuklaroglu et al., 2018).

Beta oscillations exhibit event-related desynchronization (ERD) during both actual and imagined movements, reflecting engagement of internal models for motor planning and execution (Gehrig et al., 2012; Jenson et al., 2020; Jenson & Saltuklaroglu, 2021; Pfurtscheller & Lopes da Silva, 1999). Following movement completion, a rebound or increase in beta power is typically observed—referred to as *postmovement beta rebound*—which has been associated with motor network inhibition, resetting of motor systems, and confidence in movement outcome predictions (Barone & Rossiter, 2021; Solis-Escalante et al., 2019). These oscillations are believed to arise from dynamic interactions between sensorimotor cortices and the basal ganglia, though their precise origins and functional specificity remain topics of ongoing research (Holgado et al., 2010; Jensen et al., 2005; Sherman et al., 2016). In addition to motor control, beta oscillations are increasingly recognized as supporting cognitive functions such as working memory and the maintenance of internal state representations (Engel & Fries, 2010; Spitzer & Haegens, 2017).

Beta rhythms are not monolithic. Variations in beta oscillations—including differences between low and high beta sub-bands, spatial distribution of beta desynchronization and rebound, and the occurrence of transient beta bursts—suggest that beta supports a range of motor and cognitive processes via flexible, context-dependent mechanisms (Kilavik et al., 2013; Sherman et al., 2016; Tzagarakis et al., 2010). For example, burst-like beta activity may encode prediction errors or confidence levels, while sustained beta may reflect ongoing maintenance of motor plans or cognitive states. While these dynamics have been studied, questions remain about how beta oscillations adapt across different contexts and cognitive demands—for example, their functional role in speech-related tasks that involve varying motor, auditory, and perceptual processing. Clarifying the diverse functional roles and developmental trajectory of beta oscillations is essential for advancing models of speech motor control and understanding how neural systems mature to support fluent communication.

### Beta Oscillations in Speech and Cognitive Processing

Beta oscillations play a central role in speech and language by supporting sensorimotor integration and internal modeling, both of which are necessary for fluent speech production and perception (Engel & Fries, 2010; Kilavik et al., 2013; Papeo et al., 2009; Weiss & Mueller, 2012). Beta activity not only is integral to basic sensorimotor coordination but also extends to higher level cognitive functions such as attention, anticipation, and working memory. The dynamic engagement of beta-band processes thus helps link sensory input, motor output, and cognitive control mechanisms during language use (Engel & Fries, 2010; Spitzer & Haegens, 2017).

During speech production, beta power decreases are consistently observed, reflecting engagement of motor planning and execution processes (Bowers et al., 2013; Gehrig et al., 2012). Notably, beta suppression is reliably detected prior to speech onset, underscoring beta’s role in the anticipatory neural activity required for precise temporal coordination—particularly for overt, voluntary speech production. Comparable modulation is also seen during speech perception, oral imitation, and auditory–motor mapping, further supporting the view that beta activity links linguistic and motor systems across a range of communicative contexts (Cao et al., 2022; Saltuklaroglu et al., 2018). Beta oscillations are also implicated in internal forward models, which enable the prediction of sensory consequences associated with self-generated or perceived speech actions and allow the brain to update motor commands accordingly—mechanisms applicable to both speech and broader motor behaviors (Bowers et al., 2013; Engel & Fries, 2010; Pulvermüller et al., 2006). Decreases in beta power during the processing of action-related verbs suggest beta oscillation involvement in mental simulations that bind language and motor representations (Papeo et al., 2009; Pulvermüller et al., 2006).

The role of beta oscillations extends to broader cognitive domains. They facilitate the integration of sensorimotor, syntactic, and semantic information—crucial for comprehending complex sentences and maintaining linguistic information in working memory (Lewis et al., 2016; Weiss & Mueller, 2012). Beta activity also reflects top-down control and can be modulated by attention, expectancy, and the processing of novel or unexpected linguistic stimuli (Spitzer & Haegens, 2017). Transient increases in beta power may serve to reactivate task-relevant information or help stabilize the current cognitive set to support ongoing communication. Importantly, beta-band dynamics are shaped by task demands, sensory modality, and communicative context (Cao et al., 2022; Gehrig et al., 2012). For example, sensorimotor beta modulation has been observed even during passive listening, indicating that the motor system may be automatically recruited during language comprehension. At the sentence level, beta power adapts to evolving linguistic and perceptual expectations, consistent with the ongoing updating of predictions (Lewis et al., 2016).

Although these findings underscore the diverse and flexible roles of beta oscillations in speech and language processing, much remains unknown about how these functions change and mature across development. Understanding age-related changes in beta dynamics is therefore critical for mapping the maturation of neural mechanisms underlying fluent language use.

### Developmental Changes in Beta Oscillations

Developmental changes in neural oscillatory activity—including, but not limited to, the beta band—have been well documented across childhood and adolescence, offering insights into the maturation of neural networks that support cognition and behavior (Cuevas & Bell, 2022; Rier et al., 2024; Ünsal et al., 2024). Specifically, beta power desynchronization is generally weaker and less differentiated in children compared to adults during nonspeech motor tasks (Gaetz et al., 2010; Heinrichs-Graham et al., 2018; Trevarrow et al., 2019). This pattern, together with the less prominent or absent postmovement beta rebound observed in younger children, is thought to reflect the gradual maturation of frontocentral and sensorimotor networks involved in movement planning and execution (Gaetz et al., 2010; Hao et al., 2020). In adults, beta oscillations are robustly involved in motor control—including speech production—supporting sensorimotor integration, temporal prediction, and top-down modulation (Engel & Fries, 2010; Kilavik et al., 2013). However, relatively little is known about the developmental trajectory of beta dynamics during speech-specific tasks, as most available evidence comes from nonspeech motor contexts. This gap is particularly significant, as fluent speech depends on finely tuned predictive and sensorimotor processes that continue to develop throughout childhood (Iverson, 2022; Redford, 2019). Age-related changes in these systems may be reflected in the modulation of beta activity.

Beyond its role in motor control, developmental differences in beta oscillations are also linked to improvements in broader cognitive functions such as temporal prediction, attentional allocation, working memory, and sensorimotor integration (Cirelli et al., 2014)—all highly relevant for speech and language acquisition. Recent comprehensive reviews have documented the developmental trajectories of several oscillatory bands, including both beta and alpha/mu rhythms (Cuevas & Bell, 2022; Rier et al., 2024; Ünsal et al., 2024), highlighting that oscillatory maturation is a gradual and multifaceted process. Nevertheless, the evolution of beta dynamics to support fluent, adultlike speech, or adaptation to varying communicative demands during development remains unclear. Identifying these developmental changes in beta oscillations is crucial for advancing models of speech motor control and for identifying neural signatures of typical and atypical language development.

### The Present Study

This study investigated developmental changes in beta oscillations across tasks that engage speech production (say), auditory speech perception (hear), and visual perception (see), enabling us to assess the neural contributions of motor planning, auditory–motor integration, and perceptual processing, and how these may change from childhood to adulthood. The say condition was intended to engage motor planning and auditory–motor mapping; the hear condition was designed to address speech perception and auditory–visual integration; and the see condition served as a nonauditory, nonmotor baseline. Previous work has suggested that production and perception tasks can modulate beta oscillations differently, involving both shared and distinct sensorimotor processes (Saltuklaroglu et al., 2018).

To capture the temporal dynamics of beta modulation, we analyzed oscillatory activity at three distinct time windows: immediately following the warning cue (primarily reflecting perceptual processing), prior to the Go cue (associated with anticipation and motor planning), and in the interval leading up to speech onset (reflecting final motor preparation). This design allowed us to address three central research questions: (1) Are there age-related differences in the modulation of beta oscillations across speech production, auditory speech perception, and visual perception tasks? (2) Do motor preparation and speech production demands differentially affect beta power across development? (3) Within the child group, is the degree of beta modulation associated with age, suggesting ongoing neural maturation? We hypothesized the following: (1) adults would show greater and more task-specific beta desynchronization (especially in the say condition), reflecting mature sensorimotor systems; (2) children would exhibit weaker and less differentiated beta suppression across conditions, consistent with ongoing neural development; and (3) in children, beta modulation would increase with age, reflecting developmental refinement of auditory–motor integration. By characterizing beta oscillatory activity across communicative contexts and age groups, this study aims to advance our understanding of how neural systems supporting speech and language processing—indexed by beta oscillations—may mature across development. To our knowledge, this is the first developmental study to characterize speech-specific beta-band modulation across preparation and perception within the same participants, revealing how sensorimotor–auditory integration supporting communication matures from childhood to adulthood.

## MATERIALS AND METHODS

### Participants

Participants included 28 adults (mean age = 27.8 ± 8.6 yr) and 50 children (mean age = 10.3 ± 1.7 yr) with no neurological conditions. Most participants were right-handed; however, eight were left-handed (1 adult, 7 children) and four were ambidextrous (2 adults, 2 children), based on the Edinburgh Handedness Inventory (Oldfield, 1971). All participants spoke English as their primary language. Adults performed within the normal range on a measure of nonverbal intelligence (TONI; Fopiano, 2021) and reported typical language skills. All children scored within the normal range on standardized speech, language, and cognitive assessments. The assessments for children included the *Goldman-Fristoe Test of Articulation* (GFTA-3; Goldman & Fristoe, 2015) to evaluate articulation skills, the *Wechsler Abbreviated Scale of Intelligence* (WASI; Wechsler, 1999) to provide a measure of verbal and nonverbal intelligence, the Core Language Index on the *Clinical Evaluation of Language Fundamentals—Fifth Edition* (CELF-5; Wiig et al., 2013) to provide measures of receptive and expressive language skills. After a full explanation of the study, written consent was obtained from adults and from one parent of each child participant. Oral and written assents were obtained from all children. Participants were compensated for their time. The study data were collected in parallel with identical procedures, experimental setups, and equipment at the University of Michigan and the University of Pittsburgh. All study procedures were approved by both institutions’ Institutional Review Boards.

**Table 1.** Demographic, cognitive, and speech-language assessment information

	Child			Adult		
	Mean	*SD*	Range	Mean	*SD*	Range
Age (yr)	10.3	1.7	7.3–13.2	27.8	8.6	18–45
Gender (f:m)	21:29			17:11		
TONI	108.5	10.5	77–130	101.7	11.7	83–125
GFTA	104.0	12.2	46–115			
CELF	114.3	12.6	85–140			
VIQ	112.4	10.6	89–138			
Speech Onset (ms)	478.0	146.2	232–862	470.3	96.8	221–636

*Note*. TONI = Test of Nonverbal Intelligence; GFTA = Goldman-Fristoe Test of Articulation; CELF = Clinical Evaluation of Language Fundamentals; VIQ = verbal intelligent quotient; Speech onset = median speech onset in say condition.

### Procedure

#### Experimental paradigm

**Figure 1.**
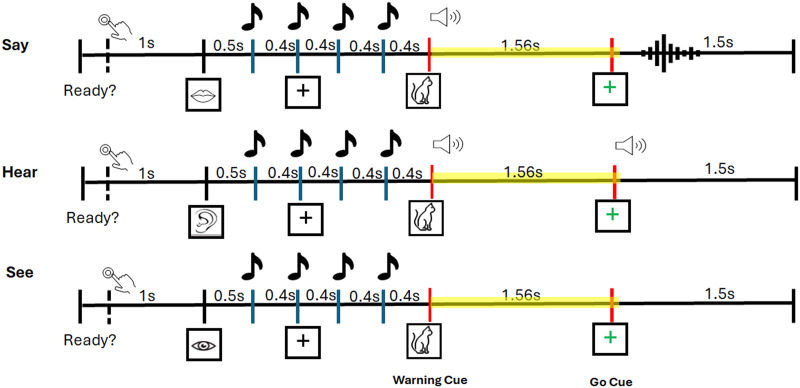
Experimental paradigm. Each trial began with a “Ready” prompt, after which participants pressed a key to initiate the trial. Following a 1,000-ms interval, a 500-ms visual cue indicated the block condition: “Say” (mouth icon), “Hear” (ear icon), or “See” (eye icon). Four rhythmic tones were then presented at 2.5 Hz. A target object appeared in all blocks for 390 ms, accompanied by a spoken word (warning cue) in the say and hear conditions. This was followed by a 1,170-ms delay, resulting in a fixed 1,560-ms interval between the onset of the warning cue and the Go cue. The Go cue was displayed for 1,500 ms, prompting participants to either speak (say), listen (hear), or passively view the screen (see). The analysis period of interest is highlighted.

Participants completed a spoken word task (Figure 1) while electroencephalography (EEG) data were recorded. They were seated approximately 140 cm from a computer monitor in a sound-attenuated booth. Each trial began with a “Ready” prompt, after which participants pressed a key to initiate the trial. Following a 1,000-ms interval, a visual cue then appeared for 500 ms, indicating the condition: “Say” (mouth icon), “Hear” (ear icon) or “See” (eye icon). This was followed by four rhythmic pure tones (1000 Hz, 100-ms duration, 400-ms stimulus onset asynchrony [SOA, 2.5 Hz rate]), delivered binaurally via insert earphones (Etymotic ER3C) at 68 dB SPL(sound pressure level). A target object (e.g., a picture of a cat) then appeared on the screen for 390 ms, accompanied by a spoken word in the say and hear conditions, functioning as a warning cue. After a fixed delay of 1,170 ms, a green cross (Go cue) appeared for 1,500 ms, prompting participants to either say the word aloud (say condition), listen passively (hear condition), or maintain fixation (see condition), consistent with the initial visual cue. The 2.5 Hz tone rate (400 ms SOA) aligns with delta-band rhythms associated with syllabic timing and prosodic structure in natural speech (Poeppel & Assaneo, 2020) and has been used to elicit temporal entrainment (Fujioka et al., 2012; Power et al., 2012). Delta activity in this range is thought to coordinate with beta oscillations to support predictive timing and auditory–motor integration (Arnal, 2012; Morillon & Baillet, 2017). In this study, rhythmic pure tones were used to establish a regular auditory rhythm and facilitate temporal expectation prior to the Go cue. While the current analysis focuses only on the rhythmic conditions, the broader protocol also included nonrhythmic conditions (tones presented in a variable, nonisochronous manner) designed to support future studies that focus on comparing the two conditions. Investigating the effects of rhythmicity was not a primary objective of the present study. All data reported here were collected for this study and have not been published or analyzed elsewhere. The fixed delay of 1,560 ms between the warning cue and the Go cue (consisting of a 390-ms warning cue plus a subsequent 1,170-ms interval) was selected to support temporal expectation while allowing sufficient time for auditory processing and preparatory activity without overlapping motor execution. Participants were instructed to listen or view passively in the hear and see conditions, with no accuracy judgments or memory checks required. In contrast, the say condition required participants to retain the spoken word across a fixed delay and prepare for articulation, engaging working memory and motor planning processes.

Each condition consisted of 35 trials, yielding a total of 105 trials across the three conditions (say, hear, see). Trials were distributed across two runs, with the order of conditions counterbalanced across participants to minimize sequence effects. In Order 1, participants completed see–say–see (Run 1), followed by hear–say–hear (Run 2); in Order 2, the sequence was hear–say–see (Run 1), followed by see–hear–say (Run 2). Each run lasted approximately 7.5 min, with brief breaks between runs. This task was part of a larger experimental protocol that included several other EEG tasks. It was administered early in the session, following an 8-min resting-state recording and a 15-min nonrhythmic variant of the current task. The total EEG session—including setup, instructions, and all experimental tasks—lasted approximately 3 hr.

Participants received standardized instructions explaining the task demands for each condition. Participants completed at least two practice trials per condition, with feedback provided by the research assistant. Additional practice was provided as needed to ensure task comprehension before beginning the main experiment. Words used in the practice trials were different from those used in the experimental trials. Procedures were consistent for both adults and children; however, younger participants were provided with additional encouragement and reminders as needed.

A research assistant was present in the EEG recording booth for the duration of the task, seated next to the participant to monitor compliance and engagement in real time. In the say condition, any disfluent, incorrect, or missing responses were flagged, though such events were rare (<2% of trials across participants) and did not warrant exclusion. In the hear and see conditions, where accuracy could not be directly assessed, participants independently advanced each trial with a button press, which helped maintain task engagement and attention throughout the paradigm. Across all conditions, the assistant monitored for signs of inattention (e.g., looking away from the screen, excessive fidgeting) and provided gentle reminders to remain focused when necessary. No trials were excluded during acquisition. All trials were retained for offline analysis, and EEG data were later screened for quality and artifacts.

#### Electrophysiological methods

EEG data were recorded using a 64-channel actiCHamp Plus system (Brain Products GmbH, Gilching, Germany) with electrodes arranged according to the international 10–20 system. Horizontal electro-oculogram (EOG) data were recorded from electrodes placed at the outer canthi of both eyes, and vertical EOG data were recorded from FP1 (as the upper vertical EOG) and an electrode placed below the left eye (as the lower vertical EOG). Data were digitized at 1000 Hz, referenced to the left mastoid, and later re-referenced offline to the average of the two mastoid electrodes. Preprocessing was performed using EEGLAB-based HAPPE pipeline (for EEGLAB, Delorme & Makeig, 2004; for HAPPE Version 3, https://github.com/PINE-Lab/HAPPE/tree/master). Electrical noise was first reduced using the multitaper regression approach implemented in the Cleanline plugin (Mullen, 2012). Data were then resampled to 250 Hz to reduce computational load and storage demands, while maintaining sufficient temporal resolution for beta-band activity. Next, data were band-pass filtered between 0.5 and 100 Hz using an ERPLAB Butterworth filter (Lopez-Calderon & Luck, 2014). The high-pass filter removed slow drifts, while the upper cutoff at 100 Hz preserved beta and low gamma activity for exploratory analysis in related projects. Bad channel rejection was carried out by combining EEGLAB’s Clean RawData functions with power spectral evaluation as implemented in HAPPE. Artifact rejection and correction for the continuous data were performed using HAPPE’s wavelet-thresholding procedures. Specifically, wavelet denoising was performed using the default Coiflet mother wavelet of order 4 (coif4), as specified in HAPPE. To further reduce residual electromyographic (EMG) artifacts, we used HAPPE’s muscle artifact rejection module, MuscIL (Gabard-Durnam et al., 2018), which applies independent component analysis (ICA) decomposition (Infomax algorithm) followed by IC classification using ICLabel (Pion-Tonachini et al., 2019). Components labeled as “muscle” with a probability of ≥25% were automatically removed, in accordance with HAPPE’s default settings, which balance artifact removal with preservation of neural signal. Data were then segmented into 8,000-ms epochs starting 5,000 ms before each warning cue. Additional artifact removal was applied using a combined approach with joint-probability criteria and amplitude-based criteria. Specifically, epochs were excluded if the signal exceeded ±150 microvolts or deviated more than 2 standard deviations from the mean, thresholds commonly used to identify nonphysiological artifacts in developmental EEG data (e.g., Gabard-Durnam et al., 2018). Bad channels were then interpolated using spherical interpolation, as implemented in the HAPPE pipeline. The average number of remaining trials included in the time-frequency analysis for each condition was as follows: 28.1 for say (children: 27.6; adults: 28.6), 29.9 for hear (children: 30.2; adults: 29.6), and 30.5 for see (children: 30.5; adults: 30.5).

#### Time-frequency analysis

Time-frequency decomposition was conducted using custom MATLAB scripts, adapted from Morales and Bowers (2022) and Cohen (2014). Spectral power in the frequency range of 0.5–50 Hz was estimated using complex Morlet wavelets convolutions, with 55 frequency steps. To optimize the time-frequency resolution, wavelet cycles were set at three cycles at the lowest frequency (0.5 Hz), increasing to 10 cycles at the highest frequency (50 Hz). Power was computed separately for all conditions for all channels, then normalized using a (dB) transform (dB power = 10 × log10[power/baseline]), with the baseline defined as the average power in the −3,000 to −2,200 ms window before the warning cue onset.

Beta power (15–25 Hz) was analyzed across three time windows: (1) P1 (postwarning cue, 300–500 ms), (2) P2 (pre-Go cue, −200–0 ms), and (3) P3 (post-Go cue, 0–200 ms), focusing on frontocentral electrodes. These were grouped into left frontal (F5, F3, FC5, FC3, C5, C3), middle frontal (F1, Fz, F2, FC1, FC2, C1, Cz, C2), and right frontal (F4, F6, FC4, FC6, C4, C6) regions (see Figure S1 in the Supporting Information, available at https://doi.org/10.1162/NOL.a.219). For statistical analyses, beta power was averaged across electrodes within each region and across each respective time window.

The 15–25 Hz range was chosen as the window for our analyses, for its established role in motor preparation and cognitive processing (Kilavik et al., 2013). While studies have used slightly different beta frequency bands between 13 and 30 Hz, the 15–25 Hz range effectively captures key motor-related beta activity while avoiding confounds from adjacent alpha (8–13 Hz) and gamma (>30 Hz) band oscillations. The selected time windows correspond to distinct stages of motor and cognitive processing, as outlined in Kilavik et al. (2013). P1 (300–500 ms postwarning cue) reflects anticipatory attention, early cognitive readiness, and initial stimulus evaluation following the warning cue (Kilavik et al., 2013). Since the warning cue consisted of a visual image in the see condition, and both a visual image and auditory word in the hear and say conditions (e.g., a picture of a cat and the spoken word “cat”), this window likely captures early sensory, lexical and semantic processing of the target object across all conditions. In the say condition, P1 may additionally reflect the onset of motor preparation in anticipation of the upcoming speech response. P2 (–200–0 ms pre-Go cue) represents peak temporal cue expectation and sustained preparatory activity. In the say condition, this period likely involves motor planning; in the hear and see conditions, it may reflect cognitive preparation and response monitoring. Beta suppression during this period has been observed even in the absence of overt movement and is thought to support expectancy, attention and sensorimotor control (Spitzer & Haegens, 2017; Tzagarakis et al., 2010, 2015). P3 (0–200 ms post-Go cue) corresponds to speech initiation in the say condition, speech perception of the auditory word in the hear condition, and sensory evaluation of the Go cue in all three conditions. During this window, beta activity may reflect continued suppression related to anticipation as well as engagement of sensorimotor networks involved in stimulus evaluation and response monitoring. Frontocentral electrodes were chosen for their relevance to motor and cognitive control processes (Pfurtscheller & Lopes da Silva, 1999). This region, encompassing sensorimotor and prefrontal areas, plays a crucial role in movement preparation and execution, making it ideal for assessing changes in beta power across these time windows (Kilavik et al., 2013; Spitzer & Haegens, 2017).

Given that these analyses focused on frontocentral regions implicated in motor and cognitive control, we did not apply spatial filtering (e.g., current source density or Laplacian transformation) or source localization techniques to address volume conduction. Thus, the results reflect scalp-level activity and should be interpreted as reflecting broader regional dynamics rather than precise neural sources.

#### Statistical analysis

Beta power was compared across conditions (say, hear, see) and between groups (adults vs. children) for each of the three time windows (P1, P2, P3). Linear mixed models were conducted using the lmer function from the lme4 package in R. Separate models were run for each time window. Each model included group, condition, and brain region (left, middle and right frontal), as well as their interactions, as fixed effects. Participant and data collection site were included as random intercepts to account for individual- and site-level variability. Linear mixed models were selected because they appropriately model repeated-measures data and allow for generalization across participants by capturing subject-level variability in beta power. Although EEG power data often deviate from normality, all power values entered into the models were normalized using a baseline-relative decibel (dB) transformation during time-frequency processing. This transformation helps to stabilize variance and reduce skew in analyses (e.g., Grandchamp & Delorme, 2011), supporting the use of parametric statistical models. Model residuals were visually inspected using QQ-plots and residuals-vs.-fitted plots to confirm approximate normality and homoscedasticity. Because our design was hypothesis-driven and focused on predefined time windows and scalp regions of interest, we used confirmatory statistical modeling rather than nonparametric cluster-based approaches typically applied in exploratory analyses.

For children, additional models explored developmental trajectories, with age, condition, and brain region (and interactions) as fixed effects, and data collection site and participant as random intercepts.

## RESULTS

### Beta Power Modulation After the Warning Cue (P1)

**Figure 2.**
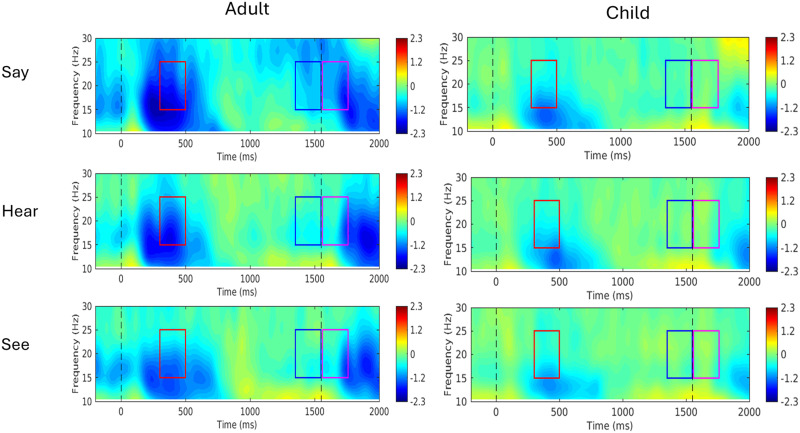
Average time-frequency over frontocentral electrodes. The boxes highlight the time windows used for beta oscillation statistical analyses (15–25 Hz): 300–500 ms after the warning cue (red box), 200 ms before the Go cue (blue box), and 200 ms after the Go cue (purple box), likely just before the onset of speech.

**Figure 3.**
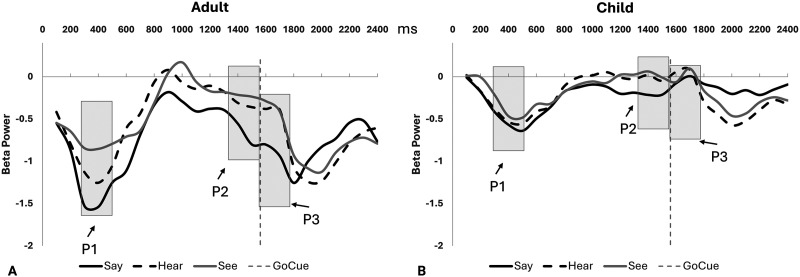
Event-related beta oscillation modulations during the three tasks, from the warning cue (time 0) to the Go cue (dashed vertical line). (A) Adults. (B) Children. Data are from frontocentral electrodes, averaged across left, middle, and right regions for visualization. Time windows: P1 (300–500 ms after the warning cue), P2 (–200–0 ms before the Go cue), and P3 (0–200 ms after the Go cue). Task conditions: say (solid line), hear (dashed line), and see (gray line). ms = milliseconds.

In both children and adults, beta power exhibited a consistent temporal trend: a decrease following the warning cue that peaked around 300–500 ms, followed by an increase later in the epoch (Figure 2 and Figure 3). In adults, beta power during the 300–500 ms window was most negative (i.e., largest suppression) in the say condition, followed by hear, and smallest in see (post hoc pairwise comparisons, *p*s < 0.05). In contrast, no significant condition effects were observed in children. The interaction between group and condition was significant (*p* < 0.001; Table 2). A significant main effect of group revealed that adults showed a greater beta power decrease compared to children (*p* < 0.001).

**Table 2.** ANOVA summary of linear mixed models comparing adults versus children across three time windows

	Degree of freedom	P1 (postwarning)		P2 (pre-Go)		P3 (post-Go)	
		*F* value	Pr(>*F*)	*F* value	Pr(>*F*)	*F* value	Pr(>*F*)
Condition (say, hear, see)	*F*(2, 608)	26.7	<0.001	32.7	<0.001	16.6	<0.001
Group (adult vs. child)	*F*(1, 76)	17.9	<0.001	9.4	0.003	34.8	<0.001
Region (left, middle and right)	*F*(2, 608)	13.8	<0.001	2.1	0.129	2.6	0.077
Condition * Group	*F*(2, 608)	10.6	<0.001	8.9	<0.001	12.9	<0.001
Condition * Region	*F*(4, 608)	1.4	0.218	0.2	0.963	0.0	0.999
Group * Region	*F*(2, 608)	7.4	<0.001	0.4	0.661	2.3	0.100
Condition * Group * Region	*F*(4, 608)	0.1	0.97	0.6	0.697	0.2	0.932

*Note*. ANOVA = analysis of variance.

Regionally, adults showed the strongest beta desynchronization in the middle frontal region, followed by the left frontal region, which in turn was stronger than the right frontal region (numeric values: middle < left < right; post hoc pairwise comparisons, *p*s < 0.05). In contrast, no regional differences were observed in children (Supplementary Table S3). A significant interaction between group and region (*p* < 0.001; see Table 2) further highlighted these group differences. When analyses were restricted to right-handed individuals, the overall regional pattern was preserved: main effects of region and the Region × Group interaction remained significant (*p*s < 0.001). Beta desynchronization was strongest in the middle frontal region (numeric values: middle < left = right), although the left–right difference was no longer statistically significant, likely due to reduced sample size. In children, no region effects were observed, consistent with the full-sample results (Supplementary Table S4).

**Figure 4.**
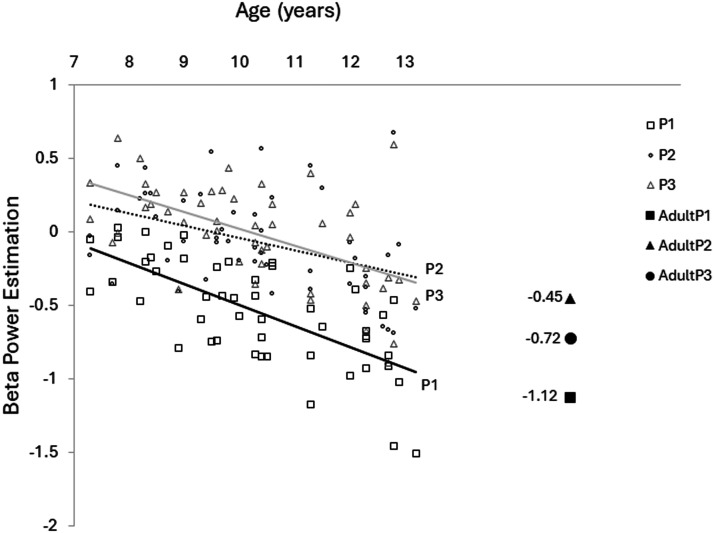
Negative correlation between beta power and age in children, shown separately for P1 (solid black line), P2 (dotted black line), and P3 (gray line). P1 represents 300–500 ms after the warning cue; P2 represents −200–0 ms before the Go cue; and P3 represents 0–200 ms after the Go cue. Reference markers indicate mean adult beta power estimates for each time window: solid square (P1), solid triangle (P2), and solid circle (P3).

In children, greater age was associated with stronger beta power suppression (*p* < 0.001; Table 3 and Figure 4), suggesting a possible developmental trajectory rather than a categorical distinction between age groups. This age-related increase in beta suppression parallels group-level differences observed between children and adults, with adults exhibiting greater beta desynchronization during task engagement.

**Table 3.** ANOVA summary of linear mixed models including age in children across three time windows

	Degree of freedom	P1 (postwarning)		P2 (pre-Go)		P3 (post-Go)	
		*F* value	Pr(>*F*)	*F* value	Pr(>*F*)	*F* value	Pr(>*F*)
Condition (say, hear, see)	*F*(2, 384)	2.8	0.059	5.4	0.005	0.2	0.825
Age	*F*(1, 48)	25.2	<0.001	6.7	0.013	14.0	<0.001
Region (left, middle, and right)	*F*(2, 384)	0.8	0.459	0.6	0.544	0.1	0.878
Condition * Age	*F*(2, 384)	2.4	0.092	1.6	0.205	0.2	0.823
Condition * Region	*F*(4, 384)	0.8	0.503	0.2	0.963	0.2	0.949
Age * Region	*F*(2, 384)	0.4	0.677	1.2	0.291	0.1	0.883
Condition * Age * Region	*F*(4, 384)	0.2	0.948	0.5	0.759	0.2	0.924

To examine whether the observed group differences in beta power modulation could be influenced by baseline activity, we compared absolute beta power between groups during the baseline period. This analysis revealed that children exhibited significantly higher baseline beta power than adults (*p* < 0.001), as well as higher beta power during the period of interest. These results suggest that group differences in dB-transformed beta suppression (i.e., ERD) may be partially influenced by differences in baseline power, but also reflect a reduced capacity for task-related suppression of beta activity in children.

### Beta Power Modulation Before the Go Cue (P2)

In the time period immediately preceding the Go cue (pre-Go), beta power again decreased in adults, with lower beta power observed in the Say condition compared to hear and see (*p* < 0.01; Figure 2 and Figure 3), though no significant difference was found between hear and see conditions. In contrast, children showed no significant condition effects. A significant Group × Condition interaction was observed (*p* < 0.001; see Table 2). Consistent with patterns observed after the warning cue, a main effect of group revealed a greater beta power decrease in adults compared to children. No significant main effects or interactions were observed across brain regions (see Table 2).

In children, increasing age was associated with a greater decrease in beta power, consistent with findings after the warning cue (Table 3 and Figure 4). Additionally, a main effect of condition revealed more beta power decrease in the say compared to the hear and see conditions (*p* = 0.005; see Table 3).

### Beta Power Modulation After the Go Cue (P3)

In the time period immediately following the Go cue (post-Go) and before speech in the say condition, beta power decreased further in adults (Figure 2 and Figure 3), with a larger decrease in the say condition compared to hear and see (*p* < 0.01), though no significant differences were observed between the hear and see conditions. In contrast, children showed no significant beta power decrease, nor any condition effect. Similar to earlier time windows, significant main effects of condition, group, as well as a Group × Condition interaction were observed (see Table 2). No significant main effects or interactions were observed across brain regions (Table 2). In children, age was negatively related to beta power (Table 3 and Figure 4), consistent with the findings for early time windows.

## DISCUSSION

This study investigated how beta oscillations support speech perception and preparation across development. Specifically, we examined beta power modulation in children and adults across three conditions—say, hear, and see—and three time windows: following a warning cue, prior to the Go cue, and prior to speech onset. Our research aimed to (1) compare beta modulation between children and adults, (2) examine condition effects and how they vary by age, and (3) assess age-related changes within the child group. Our findings reveal significant age-related differences in beta power dynamics and condition-specific modulations, offering insights into the developmental trajectory of neural oscillations involved in speech processing.

### Key Findings and Interpretation

We observed distinct patterns of beta modulation between adults and children across all time windows. Adults demonstrated significant beta power decreases, particularly in the say condition, consistent with previous research indicating that beta desynchronization supports motor preparation and sensorimotor integration (Gehrig et al., 2012; Pfurtscheller & Lopes da Silva, 1999). In contrast, children exhibited weaker beta suppression and did not differentiate the say condition from the hear and see conditions, where only say required motor preparation and working memory engagement to retain and prepare the verbal response. These findings may suggest developmental differences in the neural systems supporting motor control and cognitive processes for speech, with potential maturation in beta modulation across this age range. However, further research is needed to clarify the mechanisms underlying these differences and establish whether they reflect true developmental processes.

#### Beta modulation following the warning cue

In line with our hypothesis that this early period would primarily reflect perceptual processing, we expected that adults would show greater beta suppression in the say and hear conditions compared with see, due to the additional auditory processing demands, with the largest suppression in say because of the added requirement for subsequent motor planning. We expected children to show weaker and less differentiated suppression across conditions. The results showed that after the warning cue, both adults and children exhibited beta suppression. However, adults showed a greater reduction in the say and hear conditions compared to the See condition. This pattern may reflect increased neural engagement when auditory speech is presented, regardless of the motor demands. Specifically, the presence of auditory speech in the say and hear conditions may elicit stronger beta suppression due to both increased sensory integration demands and the automatic activation of sensorimotor networks in response to speech input (Oliveira et al., 2021). Additionally, the greater beta reduction in the say condition compared to the hear condition may reflect additive neural processes, including both sensorimotor coding of auditory input and the additional demands of preparing for speech production later in the task. Sensorimotor coding is likely engaged in both conditions, as participants process auditory cues and prepare to act; however, only the say condition requires explicit motor planning and response execution. This interpretation is supported by prior work showing that beta desynchronization reflects both auditory–motor integration and motor preparation demands in speech contexts (Bowers et al., 2013; Gehrig et al., 2012; Hickok, 2012; Weiss & Mueller, 2012). This aligns with findings that more complex tasks evoke stronger beta suppression due to increased sensorimotor demands (Saleh et al., 2010).

In contrast, children showed weaker beta modulation and no significant differences between conditions. This pattern suggests developmental immaturity in motor preparation and cognitive processes related to speech perception (Coleman et al., 2023). Although children successfully performed the tasks, the absence of condition-specific beta differences may reflect a combination of factors, including reduced neural differentiation of motor and perceptual processes and possibly lower task engagement.

In adults, beta desynchronization following the warning cue showed robust midline dominance and nominal left–right asymmetry, a pattern consistent even when only right-handed adults were included (see Supplementary Table S4). No such regional differences appeared in children, in line with previous research showing less differentiated beta modulation in younger groups (Heinrichs-Graham et al., 2018; Trevarrow et al., 2019).

Following the initial beta decrease in response to the warning cue, adults exhibited a clear beta rebound, defined as an overshoot of baseline activity, peaking around 800–1,000 ms. This pattern is consistent with prior work suggesting that beta rebound reflects postprocessing disengagement or the reinstatement of inhibitory control in motor contexts (Kilavik et al., 2013), as well as transitions in cognitive state following working memory engagement (Coleman et al., 2023). In contrast, children demonstrated only a partial rebound toward baseline, suggesting developmental differences in the recovery of sensorimotor activity after initial task engagement. This rebound may reflect a partial reset of neural activity following initial cue-related processing, even as participants remain cognitively engaged, particularly in the say condition, which involves sustained working memory demands.

#### Pre-go cue beta modulation

We hypothesized that this period, associated with anticipation and motor planning, would elicit the strongest beta suppression in the say condition for adults, followed by hear, and smallest in see, reflecting greatest motor preparation demands during say. For children, we expected weaker suppression and minimal condition differences, reflecting immature anticipatory control mechanisms. The results showed that, before the Go cue, beta power gradually decreased, with adults showing a greater reduction in the say condition compared to the hear and see conditions. Contrary to our hypothesis, hear did not differ from see, indicating that pre-Go beta suppression in adults was selectively enhanced for speech motor preparation but not clearly differentiated between auditory anticipation and passive viewing. This pattern suggests that, under the present task conditions, pre-Go beta modulation appeared to be more strongly linked to motor-related anticipatory processes than to auditory prediction mechanisms (Doyle et al., 2005; Kilavik et al., 2013). In contrast, children showed overall weaker beta reduction and no significant condition differences during this period. This suggests that while anticipatory processes may be present in children, they are likely less differentiated and less robust compared to adults. This interpretation aligns with previous research indicating that anticipatory motor networks continue to mature across childhood (Gaetz et al., 2010; Heinrichs-Graham et al., 2018; Trevarrow et al., 2019). Additionally, differences in beta suppression may also reflect developmental variation in temporal prediction abilities—adults may engage motor systems more efficiently due to better temporal expectation (Cirelli et al., 2014; Etchell et al., 2016).

The trial timeline in the present study was not jittered, and the temporal structure was highly predictable by design. This structure was used to enhance neural entrainment and to probe beta oscillatory dynamics under conditions of high temporal expectation. Prior studies have shown that predictable timing enhances beta desynchronization, particularly in adults (Etchell et al., 2016; Fujioka et al., 2012). Thus, it is possible that some of the group differences observed in our study reflect developmental variation in the ability to exploit temporal regularities. While we interpret our results primarily in terms of motor and cognitive maturation, we recognize that temporal prediction is a related and potentially contributing mechanism. Future analyses contrasting rhythmic and nonrhythmic conditions will help clarify the role of temporal predictability in shaping beta dynamics across development.

#### Post-go cue beta modulation

We predicted that this final prespeech period would show the strongest beta suppression in the say condition for adults, peaking around speech onset, with children showing weaker or absent suppression and reduced condition specificity. Results showed that following the Go cue, beta power continued to decrease in adults during the say condition, peaking around 300 ms post Go, just before speech onset (see Table 1). This beta power suppression was larger in the say condition compared to the hear and see conditions, reflecting movement preparation and initiation in the sensorimotor cortex. This finding aligns with beta suppression observed in other movement tasks (Wheaton et al., 2009), where it is linked to the transition from motor planning to execution of motor responses. In the context of speech, this suggests that adults engage domain-general motor execution mechanisms during speech production. In contrast, no significant differences were observed between the hear and see conditions, underscoring the specific involvement of beta suppression for speech motor preparation in the say condition in adults. Notably, children exhibited neither significant post-Go beta suppression nor condition differences. This absence of beta modulation may reflect developmental immaturity in the recruitment of motor execution networks during speech tasks. Developmental fMRI studies show that children gradually increase activation in motor-planning and frontal regions with age, indicating ongoing specialization in speech motor control circuits (e.g., Hashizume et al., 2014).

#### Baseline power differences

To rule out potential group-level differences in baseline activity, we conducted additional analyses of absolute beta power during the baseline period. These analyses revealed that children exhibited significantly higher beta power than adults both at baseline and during task periods. This finding suggests that the observed group differences in dB-transformed beta suppression (i.e., ERD) may be partially influenced by baseline power levels. However, because children also showed elevated beta power during the event periods, even after normalization to baseline, the reduced ERD observed in children likely reflects a combination of elevated baseline activity and reduced task-related suppression. These results support the validity of our dB-normalized comparisons and reinforce the interpretation that children engage less efficient beta suppression during speech processing.

### Developmental Trajectory of Beta Oscillations

Differences in beta power modulation between adults and children suggest a developmental trajectory in which beta oscillations become more robust and task-specific with age (Cuevas & Bell, 2022; Ünsal et al., 2024). Adults exhibit stronger, more condition-dependent beta desynchronization during speech production tasks, reflecting greater maturity and specialization of sensorimotor networks (Gaetz et al., 2010; Rier et al., 2024). In contrast, children show weaker and less differentiated beta modulation across task conditions, consistent with prior work indicating ongoing maturation of the neural systems underlying speech-related motor and cognitive processes (Iuzzini-Seigel et al., 2015; Trevarrow et al., 2019). Our findings also revealed that greater age within the child group was associated with stronger beta suppression during task engagement. This pattern aligns with previous research showing reduced beta desynchronization in children during motor tasks (Gaetz et al., 2010; Heinrichs-Graham et al., 2018) and supports the view that beta oscillations continue to develop as children transition into adolescence (Trevarrow et al., 2019). These developmental changes may reflect gradual improvements in the coordination of neural networks supporting speech planning and execution.

Alternative explanations, such as attentional variability or inconsistent task compliance, could also contribute to differences in beta suppression observed in children. While practice trials were implemented and task engagement was monitored throughout the experiment to mitigate these factors, their influence cannot be entirely ruled out. Future research incorporating independent measures of attention and compliance could help clarify their role in developmental differences in beta activity.

Nevertheless, our findings support the interpretation that reduced beta suppression in children reflects immaturity in the integration of cognitive and motor systems critical for speech processing (Cirelli et al., 2014; Smith, 2006). Beta oscillations have been linked to top-down motor control, predictive timing, and sensorimotor integration (Engel & Fries, 2010), all of which are crucial for fluent speech. Thus, the observed age-related increase in beta desynchronization may index the gradual emergence of more efficient and coordinated neural processes. This developmental interpretation provides a framework for understanding how neural dynamics evolve to support increasingly refined speech behavior throughout childhood.

### Cognitive and Motor Integration

Beta oscillations play a crucial role in linking motor and cognitive processes essential for fluent speech production, including attention, anticipation, and working memory (Arnal, 2012; Engel & Fries, 2010). In the present study, the say condition elicited the strongest beta suppression, consistent with evidence that sensorimotor beta power reflects the demands of speech motor planning and cognitive load, including working memory (Jenson & Saltuklaroglu, 2021; Piai et al., 2015; Saltuklaroglu et al., 2018). Saltuklaroglu et al. (2018) reviewed evidence that beta power decreases during overt speech production, reflecting motor preparation and execution, while Jenson and Saltuklaroglu (2021) demonstrated that beta oscillatory activity is modulated by working memory demands during syllable discrimination tasks. Together, these studies support the interpretation that beta oscillations function as a neural bridge, integrating cognitive functions—such as attention, anticipation, and working memory—with the sensorimotor processes required for fluent speech (Piai et al., 2015; Spitzer & Haegens, 2017). Our findings are consistent with the view that beta oscillations synchronize internal cognitive states with motor planning demands, particularly in tasks with substantial speech production requirements.

The reduced differentiation in beta suppression across conditions observed in children likely reflects developmental changes in neural networks supporting cognitive–motor integration, rather than a lack of speech proficiency per se. Prior research indicates that, even in middle childhood, neural systems underlying speech-related motor control and cognitive coordination continue to mature in terms of timing, consistency, and cortical recruitment (Parviainen et al., 2011, 2019; Thorpe et al., 2016). More specifically, beta oscillations have been linked to developmental changes in sensorimotor prediction and top-down modulation, supporting increasingly efficient alignment of sensorimotor prediction and top-down modulation with age. Developmental studies of limb movement show that beta-band activity and corticomuscular coherence strengthen across childhood and adolescence, supporting more efficient and predictive motor control (Spedden et al., 2019; Trevarrow et al., 2019). Although these findings derive from non-speech motor tasks, the developmental trajectory they reveal is consistent with the broader maturation of cognitive-motor integration systems and is likely relevant for speech. In speech-specific contexts, beta oscillations have been associated with top-down gating of prediction errors, further highlighting their broad role in coordinating cognitive and motor systems (Hovsepyan et al., 2023).

While children aged 7–13 are capable of fluent speech, the underlying neural mechanisms for coordinating speech planning and execution under varying demands may still differ from those of adults. The present findings thus suggest that beta activity may serve as a useful neural indicator of how flexibly and efficiently these systems are coordinated, offering insight into typical developmental trajectories and potential deviations relevant to speech-related disorders.

### Implications for Speech Disorders

These results have important implications for understanding speech disorders, such as stuttering, which involve disruptions in motor control and speech planning. Previous studies have reported altered beta power in individuals who stutter (Caruso et al., 2023; Jenson et al., 2018; Mersov et al., 2016; Saltuklaroglu et al., 2017), particularly during motor preparation for speech (Etchell et al., 2015). However, findings regarding beta oscillations in stuttering have been inconsistent. For example, Mersov et al. (2016) observed stronger beta ERD in people who stutter, interpreted as reflecting a more strongly inhibited motor system that requires greater beta suppression to initiate movement. In contrast, Jenson et al. (2018) found weaker beta ERD, which they linked to reduced sensorimotor stability. Adding further nuance, Saltuklaroglu et al. (2017) demonstrated that beta oscillations could differentiate between stuttering and nonstuttering individuals in a perceptual task, suggesting a role for beta activity beyond motor planning. Taken together, these findings suggest that atypical beta modulation in stuttering may reflect multiple underlying mechanisms—ranging from motor inhibition to deficits in sensorimotor integration—depending on task context and individual characteristics. In this context, the robust beta suppression observed in adults in our study may indicate mature speech motor control and could serve as a potential biomarker for efficient speech preparation. In contrast, weaker suppression in children may indicate developmental vulnerability in speech motor planning and execution. Future research should further investigate how task demands, developmental stages, and individual variability interact to shape beta dynamics in speech disorders.

### Limitations and Future Directions

While this study focused specifically on event-related changes in beta oscillations due to their strong association with motor control and predictive timing in speech, several limitations should be acknowledged. First, we did not analyze other frequency bands, such as alpha/mu, which also undergo significant developmental changes and are implicated in sensorimotor and cognitive processes relevant to speech (Cuevas & Bell, 2022; Rier et al., 2024; Ünsal et al., 2024). Inclusion of these bands in future work could provide broader insight into the maturation of cortical networks supporting speech and motor function. Second, our analytic approach, which targeted the beta band using wavelet-based spectral decomposition at the sensor level, does not fully exclude the possibility that alpha-band activity influenced the observed beta effects. Spectral leakage, band overlap, and cross-frequency interactions (e.g., alpha–beta coupling) may have influenced observed beta-band activity (Canolty & Knight, 2010; Cohen, 2014; Donoghue et al., 2022). While we employed wavelet-based spectral decomposition to target the beta range, our analysis did not explicitly model cross-frequency coupling or separate periodic from aperiodic components in real time. Future work using methods such as source localization, multivariate models, or phase–amplitude coupling analysis may better disentangle these effects (Cohen, 2014; Herrmann et al., 2014). Third, we did not explicitly separate oscillatory (periodic) components from aperiodic (1/f) background activity in our spectral analyses. Recent work has emphasized the importance of distinguishing between these components to accurately interpret developmental differences in neural oscillations (Donoghue et al., 2022; Rier et al., 2024). Incorporating such methods in future studies will strengthen interpretations regarding the functional role of specific oscillatory dynamics. Fourth, our experimental design used a highly predictable, nonjittered timeline to probe neural entrainment and beta modulation under conditions of strong temporal expectation. However, this temporal predictability may have disproportionately benefited adults, who are more adept at temporal prediction, thereby contributing to age group differences in beta suppression (Cirelli et al., 2014; Etchell et al., 2016; Fujioka et al., 2012). Future studies could directly compare beta dynamics in jittered versus nonjittered protocols to clarify the role of temporal expectation in development. Fifth, although engagement was monitored in real time, we did not collect independent behavioral indices of attention (e.g., catch trials), which limits our ability to fully dissociate developmental effects from residual engagement differences; we consider this as a priority for future work. Finally, because analyses were performed at the sensor level, volume conduction can both limit spatial specificity and mix functionally distinct sources (e.g., auditory vs. sensorimotor), potentially obscuring condition effects. Future work could build on the present work using source-level or information-decomposition methods (e.g., temporal ICA, generalized eigenvalue decomposition) to further isolate functionally distinct components and refine interpretability; where feasible, higher-density EEG or concurrent neuroimaging could provide complementary spatial constraints (Cohen, 2014; Donoghue et al., 2022; Herrmann et al., 2014). Together, these limitations highlight important directions for future research seeking to comprehensively characterize the neurophysiology of speech development.

## CONCLUSION

Results from this study support a developmental framework in which beta oscillations serve as a neural marker of motor and cognitive integration during speech, with implications for both typical development and speech-language disorders. This study advances our understanding of how beta oscillations develop in relation to speech perception and production. The robust beta desynchronization observed in adults during speech tasks contrasts with the weaker and less differentiated patterns in children, suggesting ongoing maturation of the neural circuits underlying speech motor planning. These findings emphasize the essential role of beta oscillations in integrating cognitive and motor processes and provide a developmental framework for future research on speech–cognition integration as well as speech-related disorders. By elucidating the neurophysiological mechanisms underpinning typical speech development, these results may inform targeted interventions aimed at improving speech outcomes in individuals with motor speech impairments.

## ACKNOWLEDGMENTS

The authors thank the children, their families, the adult participants, and the research assistants for their valuable contributions to this study and for their time and enthusiasm. We also thank the reviewers for their insightful comments and critiques, which greatly improved the manuscript.

## FUNDING INFORMATION

Soo-Eun Chang, National Institute on Deafness and Other Communication Disorders (https://dx.doi.org/10.13039/100000055), Award ID: R01DC018283.

## AUTHOR CONTRIBUTIONS

**Yanni Liu**: Conceptualization: Equal; Data curation: Equal; Formal analysis: Lead; Funding acquisition: Supporting; Investigation: Lead; Methodology: Lead; Project administration: Equal; Visualization: Lead; Writing – original draft: Lead; Writing – review & editing: Equal. **Amanda Hampton Wray**: Conceptualization: Equal; Data curation: Equal; Funding acquisition: Supporting; Investigation: Supporting; Methodology: Supporting; Project administration: Equal; Writing – review & editing: Equal. **Soo-Eun Chang**: Conceptualization: Equal; Data curation: Equal; Funding acquisition: Lead; Investigation: Supporting; Methodology: Supporting; Project administration: Equal; Writing – review & editing: Equal.

## DATA AND CODE AVAILABILITY

EEG preprocessing was conducted using the EEGLAB-based HAPPE pipeline (EEGLAB: Delorme & Makeig, 2004; HAPPE Version 3: https://github.com/PINE-Lab/HAPPE). Time-frequency analyses were adapted from publicly available code by Morales and Bowers (2022), available at https://osf.io/taed5/. Aggregated data supporting the statistical analyses reported in this study are publicly available at https://osf.io/cg4wh/.

## Supplementary Material



## TECHNICAL TERMS

Neural oscillations: Rhythmic brain activity that coordinates timing and interactions across neural networks.
Alpha/mu oscillations (8–12 Hz): Brain rhythms associated with attention, sensory gating, and motor suppression.
Beta oscillations (13–30 Hz): Brain rhythms supporting motor control, predictive timing, and speech-related sensorimotor integration.
Event-related desynchronization (ERD): A task-related decrease in oscillatory power, reflecting neural engagement or activation during processing.
Speech motor planning: Neural and cognitive processes that prepare articulatory movements before speech production.
Post-movement beta rebound: A transient increase in beta power after movement, linked to motor system resetting and inhibition.
Developmental trajectory: Age-related changes in brain or behavior reflecting neural maturation across childhood and adolescence.
Sensorimotor integration: The linking of sensory inputs and motor outputs to support fluent speech and coordinated action.
Temporal prediction: The expectation of event timing that guides perception, attention, and motor preparation.
Time-frequency analysis: An EEG method that decomposes brain signals to reveal how rhythmic activity changes over both time and frequency.
