# Supplementary material for: Speech Perception and Preparation Are Supported by Distinct Neural Dynamics Across Development
Source: Neurobiol Lang (Camb). 2026 Feb 20;7:NOL.a.219. doi: 10.1162/NOL.a.219 (PMC12978683; doi:10.1162/NOL.a.219)
Supplement: Supplementary file 1 [file nol-07-219-s001.pdf]

Supplementary Table S1. Estimated Marginal Means of Beta Power by Condition, Group, and Time Window

Group	Condition	P1 (post-warning)	P2(pre-Go)	P3 (post-Go)
		Estimate (SE)	Estimate (SE)	Estimate (SE)
Adult	Say	-1.363 (0.162)	-0.745 (0.136)	-1.005 (0.139)
Adult	Hear	-1.120 (0.162)	-0.330 (0.136)	-0.545 (0.139)
Adult	See	-0.793 (0.162)	-0.209 (0.136)	-0.531 (0.139)
Child	Say	-0.630 (0.145)	-0.195 (0.120)	-0.058 (0.122)
Child	Hear	-0.570 (0.145)	-0.027 (0.120)	-0.043 (0.122)
Child	See	-0.499 (0.145)	-0.041 (0.120)	-0.015 (0.122)

Estimates are marginal means averaged over Region (left, middle and right); standard errors in parentheses.

Supplementary Table S2. Pairwise Comparisons Between Conditions by Group and Time Window

Group	Contrast	P1 (post-warning)			P2 (pre-Go)			P3 (post-Go)		
		Estimate	SE	P-value	Estimate	SE	P-value	Estimate	SE	P-value
Adult	Say – Hear	-0.243	0.077	**0.0048**	-0.415	0.074	**<.0001**	-0.459	0.080	**<.0001**
Adult	Hear – See	-0.327	0.077	**0.0001**	-0.121	0.074	0.2276	-0.014	0.080	0.9831
Adult	Say – See	-0.571	0.077	**<.0001**	-0.536	0.074	**<.0001**	-0.473	0.080	**<.0001**
Child	Say – Hear	-0.06	0.058	0.5515	-0.168	0.055	0.0068	-0.016	0.060	0.9626
Child	Hear – See	-0.07	0.058	0.4423	0.014	0.055	0.9658	-0.027	0.060	0.8921
Child	Say – See	-0.13	0.058	0.0624	-0.154	0.055	**0.0146**	-0.043	0.060	0.7529

Degrees of freedom are constant (df = 608) for all comparisons; p-values Tukey-adjusted.

Supplementary Table S3. Estimated Marginal Means of Beta Power by Region and Group in P1 (post-warning)

Group	Region	Estimate (SE)	Contrast	Estimate (SE)	P-value
Adult	Left	-1.069 (0.162)	Left vs Middle	0.252 (0.077)	**0.0032**
Adult	Middle	-1.321 (0.162)	Left vs Right	-0.183 (0.077)	**0.0466**
Adult	Right	-0.886 (0.162)	Middle vs. Right	-0.435 (0.077)	**<.0001**
Child	Left	-0.567 (0.145)	Left vs Middle	0.032 (0.058)	0.8414
Child	Middle	-0.600 (0.145)	Left vs Right	-0.036 (0.058)	0.8076
Child	Right	-0.531 (0.145)	Middle vs. Right	-0.048 (0.058)	0.4639

Estimates are marginal means averaged over Condition (Say, hear and see); standard errors in parentheses.

Supplementary Table S4. Estimated Marginal Means of Beta Power by Region and Group in P1 (Post-warning), Right-Handed participants only

Group	Region	Estimate (SE)	Contrast	Estimate (SE)	P-value
Adult	Left	-0.987 (0.119)	Left vs Middle	0.225 (0.081)	**0.0162**
Adult	Middle	-1.212 (0.119)	Left vs Right	-0.141 (0.081)	**0.1948**
Adult	Right	-0.846 (0.119)	Middle vs. Right	-0.366 (0.081)	**<.0001**
Child	Left	-0.558 (0.102)	Left vs Middle	0.022(0.064)	0.9373
Child	Middle	-0.580 (0.102)	Left vs Right	-0.026 (0.064)	0.9147
Child	Right	-0.533 (0.102)	Middle vs. Right	-0.047 (0.064)	0.7367

Estimates are marginal means averaged over Condition (Say, hear and see); standard errors in parentheses.

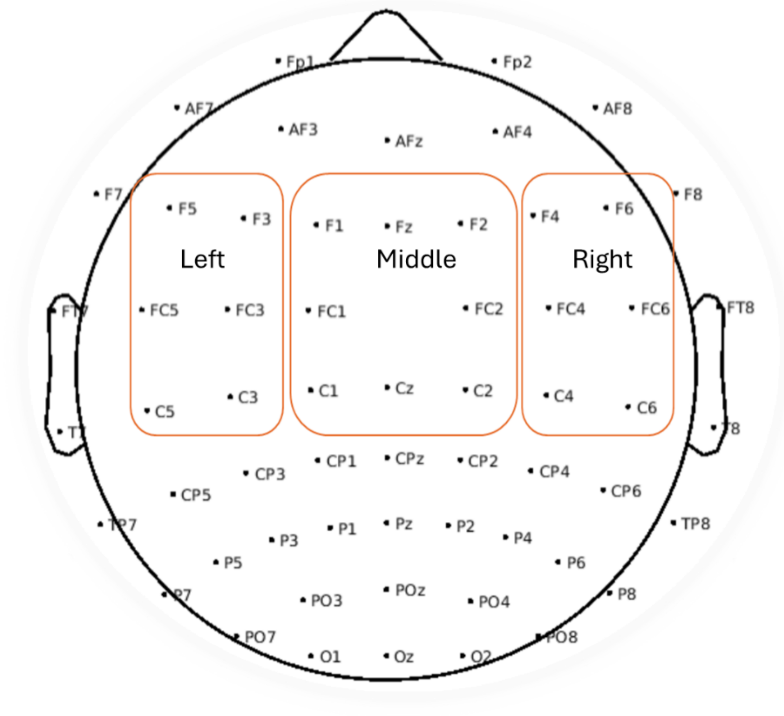

Figure S1. Schematic representation of EEG electrode groupings over frontal-central scalp regions. Electrodes are grouped into three regions of interest: Left (F5, F3, FC5, FC3, C5, C3), Middle (F1, Fz, F2, FC1, FC2, C1, Cz, C2), and Right (F6, F4, FC6, FC4, C6, C4). This regional grouping was used to examine lateralized and midline neural activity during task performance.
